# Draft genome sequence of *Aureobasidium pullulans* ATCC15233

**DOI:** 10.1128/mra.00756-24

**Published:** 2024-12-16

**Authors:** Shuang Li, Xiuyun Wu, Jing Meng

**Affiliations:** 1 The State Key Laboratory of Microbial Technology, Shandong University, Qingdao, Shandong, China; 2 Jinan Maternity and Child Care Hospital, Jinan, Shandong, China; University of Maryland School of Medicine, Baltimore, Maryland, USA

**Keywords:** genome

## Abstract

*Aureobasidium pullulans* is a well-studied polyextremotolerant generalist fungus with a ubiquitous distribution, which can efficiently secret extracellular polysaccharides, especially pullulan. Here, we reported the draft genome of *A. pullulans* ATCC15233, whose genome length is 30,444,007 bp, with a GC content of 50.63%.

## ANNOUNCEMENT

*Aureobasidium pullulans* is an oligotrophic, saprophytic, and polymorphic yeast-like fungus with ubiquitous distribution, naturally occurring in a variety of terrestrial and aquatic environments (1). The fungus is primarily used for the production of pullulan, a new food additive, with applications in the food, pharmaceutical, and medical industries (2, 3). To elucidate the pullulan synthesis and regulation mechanism of *A. pullulans*, it is of great significance to sequence and analyze the whole genome. Therefore, we report the genome sequence of *A. pullulans* ATCC15233.

*A. pullulans* ATCC15233 was purchased from the Chinese microbial bank (http://www.biobw.org/China-strain/bio-68046.html). To prepare DNA for sequencing, the seed broth included 10.0 g of sucrose, 4.0 g of K_2_HPO_4_, 2.0 g of NaCl, 1.5 g of yeast extract, 0.8 g of (NH_4_)_2_SO_4_, and 0.2 g of MgSO_4_ in 1 L of distilled water was inoculated using a single colony and incubated at 28°C for 24 h in a shaking incubator. Genomic DNA was extracted using the Fungal DNA extraction kit (magnetic beads) (Majorbio, shanghai, China) according to the manufacturer’s protocol. Purified genomic DNA was quantified and high-quality DNA was used to do further research. DNA samples were sheared into ~400 bp fragments using a Covaris M220 Focused Acoustic Shearer following the manufacturer’s protocol. Illumina sequencing libraries were prepared from the sheared fragments using the NEXTFLEX Rapid DNA-Seq Kit. The prepared libraries were then sequenced using a 2 × 150 bp paired-end configuration on Illumina Novaseq 6000 (Illumina Inc., San Diego, CA, USA). The raw data generated by Illumina sequencing (fastq format) were clipped using fastp v0.23.0 to obtain high-quality clean data (4). The reads were adapter clipped, non-AGCT bases on the 5′ ends were removed before being cut and quality trimmed (sequencing quality values, <Q20), N-containing (> 10%) reads were removed, and small fragments (length, <25 bp) were abandoned following adapter clipping and quality trimming. The high-quality reads were evaluated by K-mer analysis using Merqury software (5), indicating a genome size of approximately 30.04 Mb. The error-corrected reads were assembled with SOAPdenovo version 2.04 (6). The sequencing depth was 178.83, which was the total number of raw bases divided by the total length of scaffolds. The gene prediction was performed by Glimmer 3.02 (7), and the predicted genes were annotated through NR, Swiss-Prot, Pfam, GO, COG, KEGG, and CAZy database using sequence alignment tools such as BLAST+ v2.3.0, Diamond v0.8.35, and HMMER v3.1b2. Briefly, each set of query proteins was aligned with the databases, and annotations of best-matched subjects (E-value <10^−5^) were obtained for gene annotation. Default parameters were used for all software unless otherwise specified.

The final draft genome contains 32 scaffolds covering 30,444,007 bp and 87 large contigs (>1,000 bp), with a total length of 30,437,223 bp (the genome coverage of 99.98%). The N50 contig length is 718,803 bp, and the N90 contig length is 215,759 bp. The genome contains 10,940 protein-coding genes, 310 tRNA operons, and 64 rRNA operons, and its G + C content in the gene region is 50.63%.

## Data Availability

The *Aureobasidium pullulans* ATCC15233 whole-genome shotgun (WGS) project has the project accession JBBFKI000000000. This version of the project (01) has the accession number JBBFKI010000000 and consists of sequences JBBFKI010000001-JBBFKI010000032. The raw sequencing data have been deposited in the Sequence Read Archive (SRA) under SRA accession number SRR29825054.

## AFTER EPUB

*[This article was published on 16 December 2024 with an error in the Acknowledgments. The last line of the Acknowledgments was added in the current version, posted on 8 January 2025.]*
